# Right or Left?

**Published:** 1890-07-12

**Authors:** 


### Right or Left?

It is said to be a fact that human beings have the confirmed habit of masticating their food with the molars on the left side of the mouth. A natural habit that is, for, of course, circumstances can, and do, reverse the fact. Circumstances in this case meaning a vacuum in the molars of the left side; a vacuum which, like others, is abhorrent to nature, and which she avoids by skipping across to the right side of the mouth to perform her pulverising operations.

A curious item in the matter is that, as a rule, people obstinately deny that they masticate on the left side. Put the question to the next person you encounter, and their ready answer will assuredly confirm the above assertion. Then proceed to test the individual, as an American, writing lately in the *Boston Sunday Globe*, did, with amusing results. The only man this inquirer met during his investigations who actually deliberately chewed on both sides of his mouth was a bass singer in a church, this individual remarking that he "made it a matter of study, because he wished to wear out his teeth evenly." For the same reason the basso makes a point of changing the position of his pipe every day, he having discovered that nothing so wears out the teeth as a pipe stem. Having the preservation of his voice as a profession in view, he finds it absolutely necessary to retain the "normal shape of the mouth, so that the notes will not sound flat." The teeth, according to him, have much to do with tone in singing, acting as sounding boards; consequently, if perfect, they are able to ring out the note rounded and full. On the contrary, "if the teeth are dented or cracked, the notes are flat and harsh, as though they were rubbed against a nutmeg-grater." It is the earnest advice of the singer in question to those who wish to become as good vocalists as himself, to masticate their food on both sides while the teeth are perfect, and in that fashion preserve the balance requisite for the purpose. If the teeth be damaged or "secondhand"—(is it the case, then, that artificial teeth also decay or waste away?)—he urges the necessity of having them filled.

Curiously enough, the filling is also a matter of serious importance to singers. "Gold, when mixed with bone, is apt to lose its proverbial jingle, while silver sends back a sort of tomato-tin reverberation. Diamond filling, being hard, and hard to get, is the best, and will fling back notes like echoes of the slashing Mississippi or the giggling Minnehaha. . . . By a judicious application of diamond filling, the human voice may become as sweet as brooklet murmurs wandering through green meadows; as full and round as rivers that flow in majesty; and as deep as the echoes of the foam-crested ocean." But to obtain such miraculous results, the singer enjoins that none but diamonds of the purest and first water should be used. In these upside-down days of ours, when diamonds are no longer the rare and costly privileges of the happy few, but, instead, embellish (?) the persons of the irrepressible many, the last injunction is, perhaps, of vital importance.

The American writer to whom we have referred tells us that, in the opinion of a dentist—and his countrymen are nothing, if not dentists—it is a matter of simple insanity not to chew on each side of their jaws, turn about. For one thing, the habit of not doing so has the awkward effect of altering the shape of the mouth; in itself a powerful reason for giving the molars on the right their equal share of duty. Few people desire to possess a mouth a corner of which dangles down, anyhow, with an ungraceful droop. "Therefore," enjoins the tooth-doctor, with an earnestness born of his love for his profession, "use both sides evenly, and eat only good food."

As an extraordinary, and, probably, a little-known fact, it may be remarked *en passant* that, before any other part of the body, the teeth are said to show the first signs of lack of nourishment in cases of starvation. It would have been interesting and easy to have proved the fact in the case of the late starving exploit at the Aquarium. If such really be a genuine fact, then the experiment must have proved rather a serious one for the fool-hardy Italian, seeing it is improbable that teeth—decay once fairly set in—can be fed up again and be coaxed back to sound health.

Closely connected with the habit of masticating persistently on one side of the mouth is the practice just glanced at, almost invariably, among smokers, of holding the pipe constantly in the one corner of the month. But, in the case of the smoker, no matter how a man may contradict you, it is always in the left corner that the pipe is inserted. This may or may not have its drawbacks, but it is very certain that of every hundred, the man whose pipe is his "other self" may be instantly picked out from the ninety-nine non-smokers by doing simply that which we are forbidden by all the laws and rules pertaining to courtesy to do with regard to a gift-horse, namely, by "looking him in the mouth," and observing his hanging lower lip—the pipe's work. On the other hand, we are told that "the mouth of a cigar smoker begins to run away to his ear," or, if he smoke a thick cigar, matters become, in time, still more complicated, for the corners of that mouth of his get lost, absorbed, mixed up in the aperture. In either case a melancholy prospect. All of which facts go to pile up the proofs that there is, indisputably, a right way and a wrong way to do all things in this life-journey of ours, even to eat a mutton chop.

Trifling! trifling! says the busy man of business, angered at his time being wasted by such a frivolous argument, and obstinately positive, in his own mind, that he, at least, chews his food on the right side. A truth, doubtless, seeing that, in his estimation, whichever side he uses must perforce be right. And his hungry brother-man, gaunt and pinched by poverty's tight grasp, agrees with him, and jeers at the, to him, mocking advice that he should avoid heaping all the work on one side of his mouth, or that he should be careful not to bite off more than he is able to masticate, always remembering that his jaws need rest as well as the other parts of his body. To him it is verily a bad form of joking, and even worse than trifling.

But is not our little span made up of just such trifles? asks "the chiel" who goes in and out among his fellow-men "taking notes." Work of all kinds must necessarily increase the size of the performing part; witness the fact that the right hand of the last President's wife increased perceptibly, owing to the enormous amount of hand-shaking to which she was subjected by Brother Jonathan and Brother Jonathan's better-half, during her graceful reign at the White House. Indeed, it is seriously asserted that her gloves had to be made separately—a large right and a small left.

Thus, by and by, when the jaw becomes enlarged on one side, and the corner of the mouth tries to travel down to the neck, the scoffer will perhaps confess there is something in it after all, and will condescend to give the matter due and proper attention, whereby he will reap a fitting reward in that nice adjustment of the balance of things of even such small importance as the human mouth.

---

### BEFORE THE LORDS' COMMITTEE.

A further instalment of the evidence has been unavoidably held over until next week.

